# Influence of home chaos on preschool migrant children’s resilience: A moderated mediation model

**DOI:** 10.3389/fpsyg.2023.1087710

**Published:** 2023-02-28

**Authors:** Jinghui Zhao, Haiyan Cui, Jing Zhou, Limin Zhang

**Affiliations:** Department of Early Childhood Education, School of Education, Guangzhou University, Guangzhou, China

**Keywords:** preschool migrant children, home chaos, family resilience, social support, children’s resilience

## Abstract

Increasing attention has been drawn to the development of preschool migrant children’s resilience recently. Resilience refers to the positive internal strengths and qualities of individuals in adverse situations, and is an essential psychological quality for preschool migrant children to cope with adversity. Home chaos as a risk factor, has an important impact on the development of individual’s resilience, but the specific mechanisms under which home chaos works have yet to be explored, especially for preschool migrant children. Based on resilience model theory, 3,135 preschool migrant children and their families were surveyed and a moderated mediating effect mode was constructed to test the effect of home chaos on preschool migrant children’s resilience. The results showed that after controlling for gender and age, home chaos significantly and negatively predicted preschool migrant children’s resilience. Family resilience played a mediating role in the relationship between home chaos and preschool migrant children’s resilience. Meanwhile, social support positively moderated the mediating effects of family resilience. The findings of this study suggested that low home chaos was conducive to promoting family resilience, which in turn fostered children’s resilience, and that social support could play its protective role in weakening the negative effects of home chaos and this had certain guiding implications for the development of resilience in preschool migrant children.

## Introduction

With the continuous advancement of China’s new urbanization process and urban–rural integration development, a large number of rural population are migrating and gathering in cities and towns, the “family-oriented” pattern of population mobility is increasingly prominent (Zhang, 2008). In China, rural-to-urban migrant children (labeled as “migrant children”) are those who are under the age of 18 and have shifted from rural residence to urban cities following their parents for at least 6 months (Chen et al., 2019). The results of National Bureau of Statistics (2021) show that the size of China’s migrant population reached 376 million people. According to statistics, the number of migrant children was 71.09 million, which was about double that of 35.81 million in 2010. Correspondingly, the number of migrant children aged 0–5 years was 11.47 million. It can be seen that preschool migrant children have become a group of migrant children which could not be ignored. Migrant children belong to disadvantaged and vulnerable groups in the city (Morris et al., 2018), and are prone to psychological problems such as loneliness, low self-esteem, depression, and a lower sense of well-being compared to non-migrant children (Simsek et al., 2021; Chang et al., 2022). However, some studies have found that the level of psychological development of migrant children shows obvious individual differences, and there are also some migrant children whose mental health level has not decreased significantly (Ren and Treiman, 2016). For this situation, more and more researchers have begun to incorporate resilience into the study of migrant children’s psychological problems, and regard it as an important factor affecting the mental health level of migrant children (Tam et al., 2020; Solà-Sales et al., 2021). Resilience refers to the ability of individuals to promote successful adaptation and sound development in the face of pressure or adversity and is a key indicator to measure the level of children’s mental health (Rutter, 2007). Migrant children with a high level of resilience are able to carry out good self-regulation in stressful situations, relieve negative emotions, and successfully cope with the negative impact of “migration” (Tam et al., 2020; Lackova Rebicova et al., 2021). And low resilience children in the growth process are easy to fall into solitude, low self-efficacy, inferiority, sensitivity, and low motivation, which seriously affect their healthy psychological development (Jiang et al., 2022). The Preschool years are an important period for the development of resilience in migrant children, and the early development of resilience is critical for the individual’s future psychological and behavioral development, academic achievement, and social competence (Masten and Barnes, 2018; Yoon et al., 2022). Therefore, it is of great research value and significance to pay attention to the development of preschool migrant children’s resilience, explore its influencing factors and mechanisms, and provide targeted suggestions for the improvement of preschool migrant children’s resilience.

After preschool migrant children move to cities, their family environment has undergone dramatic changes and has affected children’s development (Kuyvenhoven et al., 2022). The development level of preschool migrant children’s resilience is not only affected by family environment factors to a large extent, but also related to risk factors and protective factors in the family (Martinez-Torteya et al., 2009). Home chaos, as an important aspect of the family physical environment, and also a risk factor in the family, has a significant impact on the development of preschool migrant children’s resilience (Evans and Wachs, 2010). Home chaos may be expected to affect children’s development through direct or indirect ways (Fiese and Winter, 2010). Direct effects on children include causing physiological reactions (Brown et al., 2019), disturbing children’s attention, and hindering the development of children’s executive function and self-regulation, and so on (Chatterjee et al., 2015; Vernon-Feagans et al., 2016). Indirectly, the over-stimulation and unpredictable character of chaotic families may be detrimental to promoting good communication and exchange between families, affecting the quality of parent–child interactions, etc., through these intra-family factors, which in turn affect children’s development (Mills-Koonce et al., 2016). However, few studies have explored the influence of home chaos on preschool migrant children’s resilience and its mechanism. Under the framework of resilience model theory, this study focuses on the family field and integrates the relationships among home chaos, children’s resilience, family resilience, and social support. And further reveal the possible mediating or moderating roles of family resilience and social support in the relationship between home chaos and children’s resilience, so that to provide targeted suggestions for cultivating and promoting preschool migrant children’s resilience.

## Literature review and hypotheses

The formation and development of children’s resilience is influenced by various factors. The theoretical model of resilience points out that children’s resilience is the process by which risk factors are counterbalanced with protective factors during their growth (Masten and Barnes, 2018). Risk factors are factors in the environment that negatively affect an individual’s survival and development. Although risk factors are conditions that stimulate an individual’s resilience, they moreover increase the likelihood of adverse adaptive consequences and play a negative role. Protective factors are factors that can contribute to better coping with life events and reduce negative development in individuals and play a positive role. Children face numerous risk factors and protective factors in their lives, especially in the family system. In recent years, a number of studies have focused on the influence of family functions, parenting concepts and parenting styles, and parent–child relationships in the family environment on children’s resilience (Tamura, 2019; Wu et al., 2020; Bruno et al., 2023). In addition, there is also a portion of studies that have examined the impact of social support in family and social networks on family and individual development (Hassanein et al., 2021). However, most of these studies have focused only on protective factors in the family, relatively neglecting the impact of the risk factors of home chaos on children’s resilience in preschool migrant children. Home chaos as a risk factor may have a negative impact on individual survival and development; family resilience and social support as protective factors could bring positive effects and motivate individuals to better cope with life events and reduce negative development. However, the interrelationship between these factors and how they work together in preschool migrant children and affect their development of resilience needs to be further investigated.

### Home chaos and children’s resilience

Home chaos, as a physical characteristic of family environment, is a key indicator to measure whether the family environment is good and suitable for children’s growth. It reflects the crowding degree, noise level and organization of the family environment, including noisy, chaotic and irregular life. It is a tangible, can be concretely perceived family subsystem (Evans and Wachs, 2010). Owing to the restriction of economic conditions, migrant families usually rent houses in the suburbs or old urban areas of migrant cities for temporary residence, and the housing quality is generally poor, such as narrow and crowded living areas, noisy surroundings, poor living facilities, etc., which is often accompanied by high home chaos (Simsek et al., 2021; Kuyvenhoven et al., 2022). At present, due to the impact of the COVID-19 pandemic, the external environment has changed greatly. The school suspension, home isolation, and economic shock brought by the epidemic have significantly increased the home chaos (Johnson et al., 2022). Living in a noisy and chaotic family environment for a long time will directly or indirectly affect the development of cognitive ability, executive function, social emotion, and mental health of migrant children (Martin et al., 2012; Raver et al., 2015; Berry et al., 2016). Previous studies have also shown that home chaos, as a risk factor, is significantly correlated with psychological performance (Zhang, 2022). Based on the above, this study puts forward the following hypotheses:

*H1:* Home chaos has a negative predictive effect on the resilience of preschool migrant children.

### Family resilience as a mediator

A question to be further explored when assessing the impact of home chaos on children’s resilience is how families respond to this situation of chaotic environment. Some researchers have noted that the important role of family resources in children’s resilience in various adversity and to link family resilience to children’s mental health (Herbell et al., 2020). Family resilience refers to a positive behavior mode and strategy exhibited by family members in response to adversity and stress that enables the family to quickly emerge from the crisis situations, and ensure the play of family functions and the development of family members (Patterson, 2002). On the one hand, family resilience may be negatively affected by home chaos. Some studies have highlighted that the accumulation of internal and external pressures can overwhelm families and increase the risk of negative outcomes (Emond, 2020). As a risk factor, home chaos will affect the overall family structure and internal resilience system, and affect the functional structure of the whole family (Marsh et al., 2020). On the other hand, family resilience can positively predict individual’s resilience. The way a family deals with adversity will affect the coping ability and adaptation of individual members (Chen et al., 2021). A chaotic family environment may impede the transmission of family patterns and beliefs, which affects children’s resilience by influencing family resilience in the family psychic environment (Daniels and Bryan, 2021). Based on the above, home chaos, as an important factor of family physical environment, may affect children’s resilience by affecting family resilience in the family’s psychic environment. However, few studies have delved into the relationship between family resilience in terms of home chaos and preschool migrant children’s resilience. Hence, this study proposes the hypothesis:

*H2:* Family resilience mediates the relationship between home chaos and preschool migrant children’s resilience.

### Social support as a moderator

Social support is the multiple forms of help or support that individuals or groups receive from their social networks (e. g., parents, peers, neighbors, communities, government, etc.; Krahn, 1993). Social support, as a protective factor of individual and extra-family systems, could play a buffer role between risk factors and their negative development outcomes (Hasan Reza and Henly, 2018), reduce the negative impact of risk factors on individual and family development, and have a protective and supporting function for individual development (Hassanein et al., 2021). It has been shown that social support acts as a positive resource with a reinforcing and buffering effect, reducing the negative emotions and reactions triggered by stressful events (Hill et al., 2021). Social support has a certain impact on family resilience (Wong et al., 2019). In the disadvantaged condition of high home chaos, family resilience may still show better results if they receive higher social support. It suggests that social support may mediate the effect of home chaos on family resilience. Therefore, this study predicts the moderating effect of social support on the relationship between home chaos and family resilience, and proposes the following hypotheses:

*H3:* Social support positively moderates the mediating effects of family resilience in the relationship between home chaos and family resilience.

### Present research

**Figure 1:**
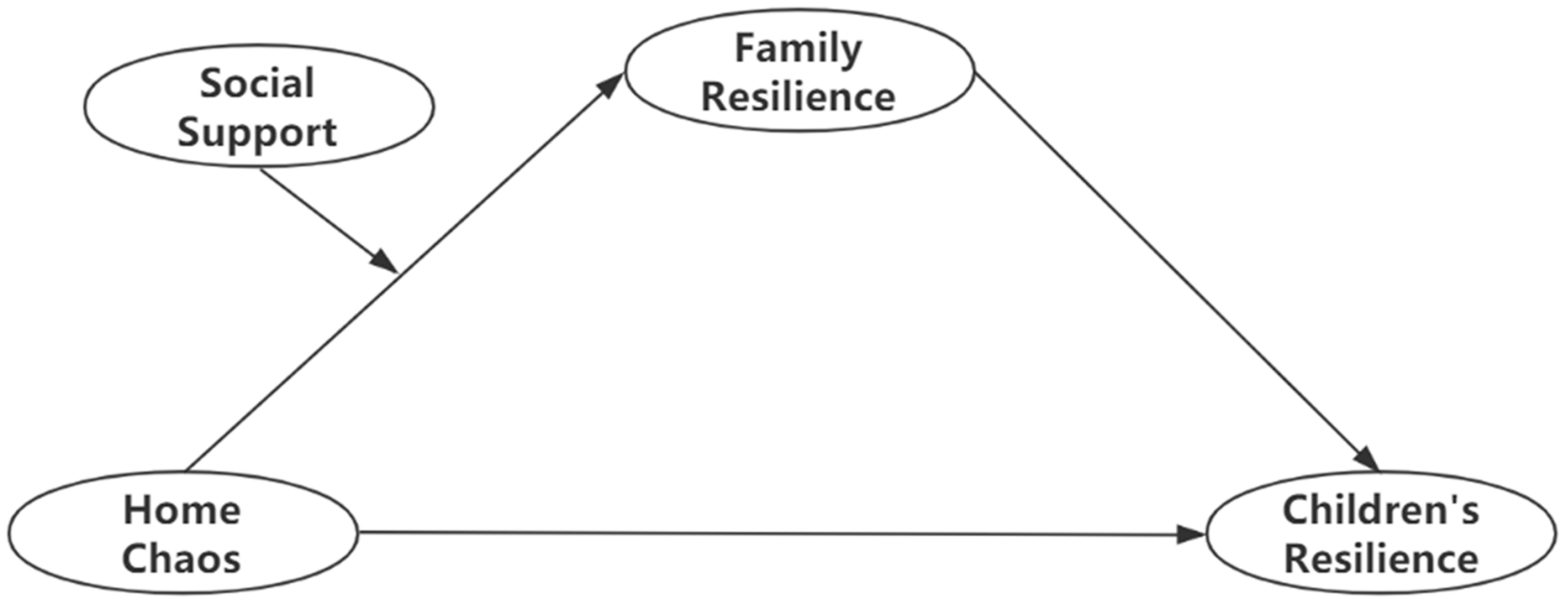
Hypothetical model.

This study took preschool migrant children as the research objects, based on the theory of resilience model, developed a moderated mediation model (see Figure 1). The present study examined the relationship and the mechanisms of action between home chaos and preschool migrant children’s resilience. And further investigated the mediating role of family resilience in the influence of home chaos on migrant children’s resilience, and the moderating role of social support.

## Materials and methods

### Participants

The current study used the convenience sampling method to select preschool migrant children and their parents from kindergartens in Guangzhou, Shenzhen and Foshan City, Guangdong Province as the research object. The questionnaires were delivered and completed through an online crowdsourcing platform^1^ in China. Before data collection, participants’ consent was acquired and all replies were anonymous. If it took less than 100 s to complete the questionnaires and answers regularly, such as the same score in each item, it was considered as an unqualified sample. After excluding the invalid samples, a total of 3,135 valid questionnaires with an effective response rate of 91.80% from 3,415 primary questionnaires were collected. The samples comprised 1,654 boys (52.8%) and 1,481 girls (47.2%). Among the children, 26.5% were 3–4 years old, 35.9% were 4–5 years old, and 37.6% were 5–6 years old. Specific demographic information was shown in Table 1.

**Table 1:** Demographic characteristics of participants (*N* = 3,135).

Statistical variables	Group	Frequency number	Effective percentage (%)
Parents	Father	671	21.4
	Mather	2,464	78.6
Age	Under 25 years	136	4.3
	26–30 years	904	28.8
	31–35 years	1,410	45.0
	36–40 years	520	16.6
	41–45 years	138	4.4
	over 45 years	27	0.9
Gender of child	Boys	1,654	52.8
	Girls	1,481	47.2
Age of child	3–4 years	831	26.5
	4–5 years	1,124	35.9
	5–6 years	1,180	37.6

### Measures

#### Home chaos scale

A version of the Confusion, Hubbub and Order Scale (CHAOS) compiled by Matheny et al. (1995) was employed in this study. It consists of 15 items which are rated on a four-point Likert scale. Items assess the extent to which the daily home atmosphere is characterized by lack of routine, confusion, and noise. Seven items reflect routines and organization (e.g., “First thing in the day, we have a regular routine at home”) and eight items reflect disorganization, confusion, and noise (e.g., “There is often a fuss going on at our home”). The routines and organization items were reverse coded before data analysis. This total score reflects the extent of home chaos, with higher scores representing more disorganized, confused, and noisy home environments. The scale has good reliability and validity in previous study (Andrews et al., 2021). The Cronbach’s alpha for this scale was 0.81 in the current study.

#### Family resilience assessment scale

This study used the revised Chinese version of the Family Resilience Assessment Scale (C-FRAS) compiled by Sixbey (2005). The revised version of the scale was tested to have a good reliability and validity (Dong et al., 2018). The scale consists of 44 items that are graded on a four-point rating scale. Considering the differences between religious issues in China and foreign countries, the C-FRAS scale was divided into four sub-dimensions which were suitable for China’s national conditions and almost identical to the original one; Family Communication and Problem Solving (e.g., “We consult with each other about decisions”), Utilizing Social and Economic Resources (e.g., “We feel people in this community are willing to help in an emergency”), Maintaining a positive attitude (e.g., “We feel we are strong in facing big problems”), and Conferring Adversity Significance (e.g., “We accept stressful events as a part of life”). A higher total score indicates a higher level of family resilience. In this study, Cronbach’s alpha were 0.92, 0.76, 0.78, and 0.71 for the four dimensions of family communication and problem-solving, utilization of socioeconomic resources, maintaining a positive attitude, and conferring adversity significance, and 0.94 for the total scale.

#### Children’s resilience scale

The Devereux Early Childhood Assessment for Preschoolers Second Edition (DECA-P2; LeBuffe and Naglieri, 2013) was used to measure children’s resilience. This scale includes three protective factor sub-scales: initiative (e.g., “Try or ask to try new things or activities”), self-regulation (e.g., “control his/her anger”) and attachment/relationship (e.g., “show affection for familiar adults”) and behavioral problem screening scales related to resilience. Only three protective factor sub-scales were used in this study. Each sub-scale contains nine items, totaling 27 questions. Using five points to score, the total score of the scale is calculated to obtain a composite resilience value with higher scores indicating higher levels of resilience. The study was scored on the primary caregivers of prechool migrant children. The scale has good reliability and validity in previous study in China (Ji et al., 2015). The Cronbach’s alpha for the three dimensions of initiative, self-regulation, and attachment/relationship were 0.85, 0.86, and 0.79, and the Cronbach’s alpha of the total scale was 0.92.

#### Perceived social support scale

Perceived Social Support Scale (PSSS), which was formulated by Zimet et al. (1988) was selected as indicators for measuring social support. It includes 12 items that are divided into three sub-dimensions, i.e., family support (e.g., “My family really tries to help me”), friend support (e.g., “I can talk about my problems with my friends”), and other significant support (e.g., “There is a special person who is around when I am in need”). Each subdimension contains four items rated on a seven-point scale. The higher the score is, the higher the perceived social support. The scale has good reliability and validity in Chinese context (Wang et al., 2017). In this study, the Cronbach’s alpha of family support, friend support, and other support dimensions were 0.85, 0.84, and 0.83, and the Cronbach’s alpha of the total questionnaire was 0.91.

#### Demographic covariates

Parents reported child’s age (1 = 3–4 years old, 2 = 4–5 years old, and 3 = 5–6 years old), and gender (0 = girl,1 = boy). Both were included as covariates.

### Statistical analysis

Data were analyzed with SPSS24.0, included reliability analysis, common method bias test, descriptive statistics, and correlation analysis. Structural equation model tests were performed by Mplus8.3. In the analysis of moderated mediation effects, the measures for all latent variables were standardized (*Z* score) to reduce multicollinearity. At the same time, in order to solve the problem that the latent variable contains many observed indexes, according to the suggestion of Matsunaga (2008), this study created item parceling by using factorial algorithm and internal consistency approach. Among them, home chaos was classified into three items by factorial algorithm, and the remaining variables were parceled according to dimensions by the internal consistency method to form new indicators of each latent variable. The maximum likelihood method (ML) was used to estimate model parameters. Due to the large sample size in this study, the chi-square values were not considered as a reference for model fit. To assess the goodness of the fit of the model, the following fit indices were chosen, such as Comparative Fit Index (CFI), Tucker-Lewis index (TLI), Standardized Root Mean Square Residual (SRMR), and Root Mean Square Error of Approximation (RMSEA). The cutoff values of CFI and TLI ≥ 0.9, SRMR and RMSEA ≤0.08 were adopted as the good fit criteria in this study. And *p* value (*p*) < 0.05 was considered as statistically significant (Kline, 2005).

## Results

### Common method bias

An unrotated exploratory factor analysis was performed on all variables, using Harman’s single-factor test (Podsakoff et al., 2003). The results showed that 16 factors had characteristic roots greater than 1. The variance explained by the first factor was 20.04%, which was lower than the critical value of 40%, indicating the absence of substantial common method bias.

### Description statistics and correlation matrix

Table 2 presents the descriptive statistics (means and standard deviations), and correlations for the main study variables. As can be seen, home chaos was negatively associated with family resilience and children’s resilience in migrants (*r* = −0.46, *p* < 0.01; *r* = −0.35, *p* < 0.01, respectively). Family resilience was positively associated with children’s resilience (*r* = 0.31, *p* < 0.01). In addition, social support was negatively associated with home chaos (*r* = −0.43, *p* < 0.01), while was positively associated with family resilience and children’s resilience (*r* = 0.51, *p* < 0.01; *r* = 0.39, *p* < 0.01, respectively).

**Table 2:** Descriptive statistics and correlation matrix for each variable (*N* = 3,135).

	1	2	3	4	5	6
1. Gender	–					
2. Age	0.02	–				
3. Home chaos	0.01	−0.01	–			
4. Family resilience	0.01	−0.04^*^	−0.46^**^	–		
5. Children’s resilience	−0.07^**^	0.05^**^	−0.35^**^	0.31^**^	–	
6. Social support	0.01	−0.04^*^	−0.43^**^	0.51^**^	0.39^**^	–
*M*	0.53	1.11	2.20	2.92	3.33	4.92
SD	0.50	0.79	0.27	0.20	0.53	0.80

^*^p < 0.05, ^**^p < 0.01, ^***^p < 0.001.

### Testing for the mediating role of family resilience

This study tested the mediating effect of family resilience based on the test procedure of mediation analysis of structural equations, and estimated confidence intervals for each coefficient by Bias-Corrected Bootstrap method (Bootstrap = 5,000), and the 95% confidence intervals that do not contain 0 indicate statistical significance (Shrout and Bolger, 2002). First, the direct effect of home chaos on migrant children’s resilience was examined, and the results showed a good model fit with RMSEA = 0.05, CFI = 0.98, TLI = 0.98, and SRMR = 0.02. After controlling for gender and age, home chaos negatively and significantly predicted migrant children’s resilience (*b* = −0.51, *p* < 0.001), and the amount of variance explained by children’s resilience was 17.7%, and hypothesis *1* was supported.

Second, adding family resilience as a mediating variable to the original model showed the same good fit, with various fit indices of RMSEA = 0.06, CFI = 0.97, TLI = 0.96, and SRMR = 0.03, with 30 and 20.4% of the variance explained by family resilience and migrant children’s resilience, respectively. Home chaos negatively significantly predicted family resilience (*b* = −0.65, *p* < 0.001) with a 95% confidence interval of [−0.72, −0.58], and family resilience significantly positively predicted migrant children’s resilience (*b* = 0.19, *p* < 0.001) with the 95% confidence interval was [0.13, 0.24], indicating that a mediating effect holds. The mediating effect size was −0.12, *p* < 0.001, with 95% confidence intervals of [−0.16, −0.09], none of the 95% confidence intervals contained 0, indicating statistical significance, and the proportion of the mediating effect to the total effect (−0.50) was 24%. Thus, family resilience partially mediated the effect between home chaos and migrant children’s resilience. Hypothesis *2* was supported.

### Testing of moderated mediation model

This study used latent moderated structural equation (LMS) to test the moderated mediation effect (Klein and Moosbrugger, 2000). Since the LMS method does not provide a traditional fit index, this study tested the model following the two-procedure steps proposed by Maslowsky et al. (2015). In the first step, a benchmark model without interaction terms was constructed, which adds the main effect of social support based on the mediation effect model of home chaos affecting children’s resilience through family resilience. The analysis showed a good fit of the benchmark model without interaction terms: RMSEA = 0.08, CFI = 0.92, TLI = 0.91, SRMR = 0.06, AIC_0_ = 95997.41, and Loglikelihood_0_ = −47952.71.

In the second step, the latent interaction term (home chaos × social support) is added to the benchmark model to form the full model. Two methods were used to determine whether the model containing the interaction terms fitted better than the benchmark model. The first method is judged by the AIC value, and if the AIC value is smaller or unchanged, the model containing the interaction term has not broken. The second method uses the Log Likelihood test, calculates the value of D = −2[Log Likelihood_0_ − Log Likelihood_1_], according to the H_0_ value, and performs the chi-square test on the results, if significant, indicating that the moderated mediation model has a better fit. Results display, AIC_1_ = 95957.61 < AIC_0_, it was decreased by 39.80, showed that the improvement of the full model. Log Likelihood_1_ = −47931.81 > Log Likelihood_0_, it increases by 20.90, so D = −2[Log Likelihood_0_ − Log Likelihood_1_] = 41.80. Taking the difference between the free parameters of the two models as the degree of freedom = 1, the chi-square test showed *p* < 0.001, the difference is significant, indicating that the moderated mediation model is better than the benchmark model. Based on the above information, it is considered that the model fit with the interaction term is acceptable, and the moderated mediation effect can be analyzed.

**Figure 2:**
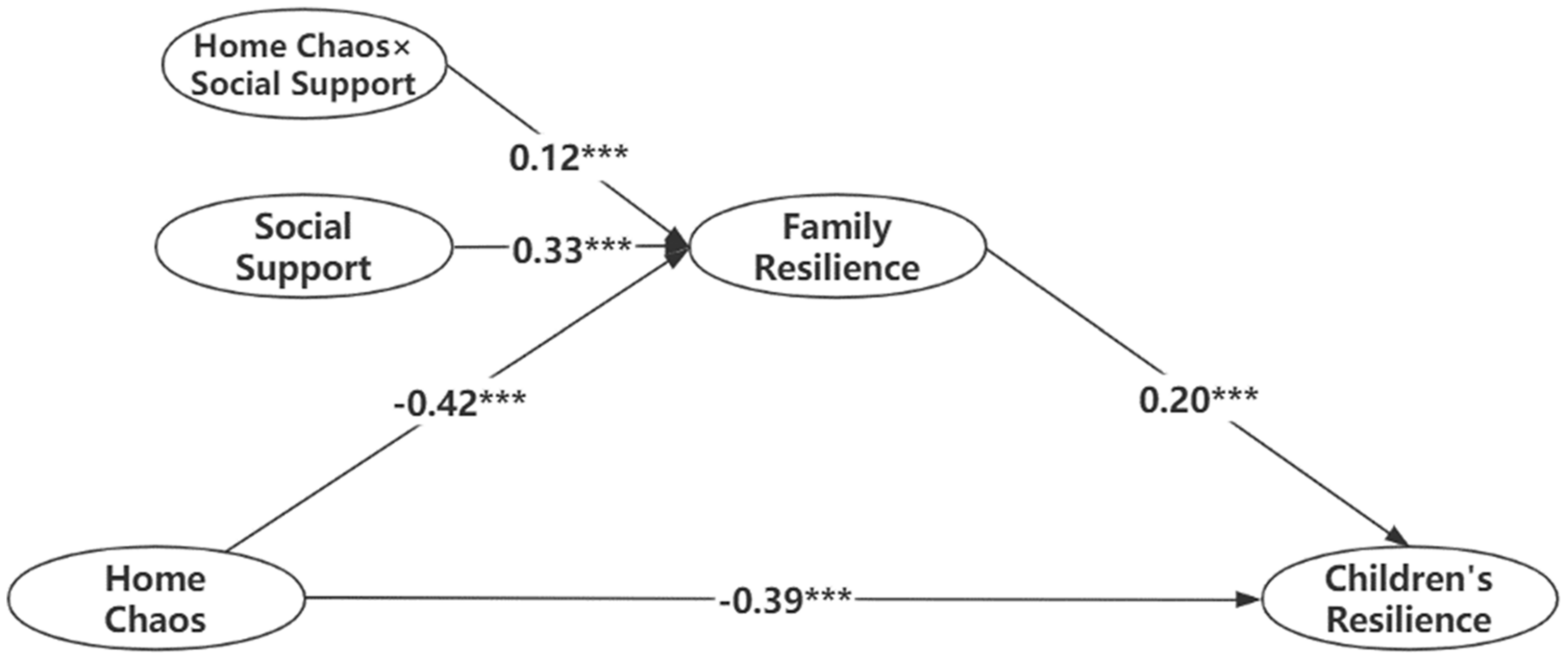
Moderated mediation analysis results. ^*^p < 0.05, ^**^p < 0.01, and ^***^p < 0.001. Gender and age were control variables, which are not shown in figure, for concise purposes.

The results of the moderated mediation model test showed that the interaction term of social support and home chaos can significantly positively predict family resilience (*b* = 0.12, *p* < 0.001). 95% confidence intervals is [0.08, 0.15]. It suggests that social support positively moderated the mediating effects of family resilience. *Hypothesis 3* is supported. Figure 2 shows the findings.

**Figure 3:**
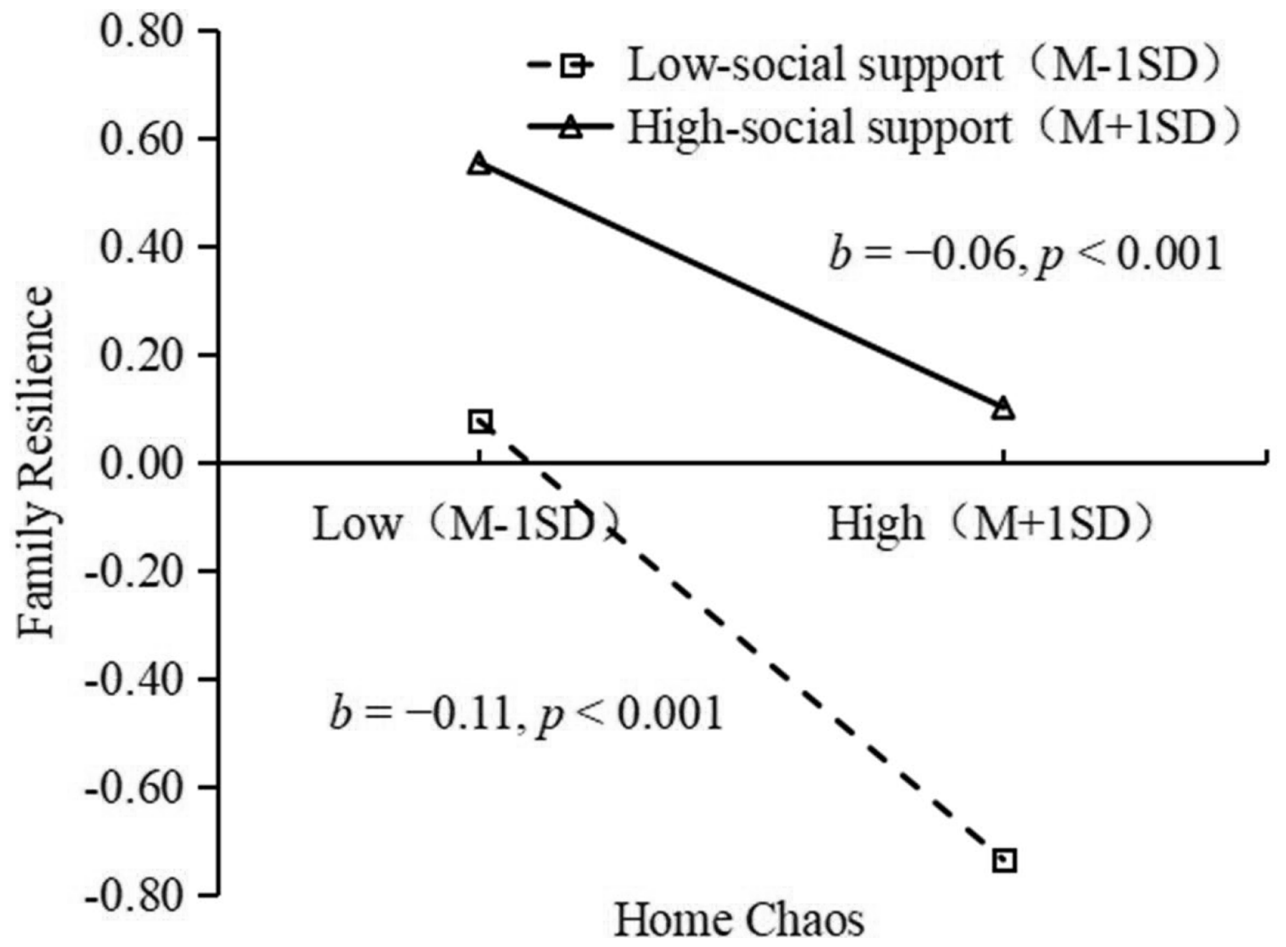
Relationship between home chaos and family resilience at different levels of social support. ^*^p < 0.05, ^**^p < 0.01, and ^***^p < 0.001.

To reveal the moderate effects more clearly, a simple slope test was performed (Dearing and Hamilton, 2006). The results show that home chaos has a significant negative prediction effect on family resilience in the low social support group (*b* = −0.11, *p* < 0.001). In the high social support group, home chaos had a significant negative prediction effect on family resistance (*b* = −0.06, *p* < 0.001). As is seen from Figure 3, as home chaos increased, family resilience in the low social support group decreased significantly, while the decreasing trend of family resilience in the high social support group slowed down, and families resilience in the high social support group was always higher than that in the low social support group. It indicated that social support played its buffering role and moderated the negative effect of home chaos on family resilience.

## Discussion

### The relationship between home chaos and children’s resilience

The results of this study suggest that home chaos had a negative effect on the development of migrant children’s resilience. High home chaos is often accompanied by high levels of stress and environmental stimuli, which are manifested as high noise level, narrow living space, chaotic daily life, lack of routine, unpredictability, and other characteristics. It is easy to overload the individual senses and attention, affect the individual’s cognitive ability and emotional state, lead to stress and physiological arousal, and have a negative effect on the individual’s mental health development (Coldwell et al., 2006). Such negative effects include: reduced frustration tolerance, judgment errors, attention, and adaptive response ability (Hong et al., 2021). Preschool migrant children experience disorganization, poor routines, or unpredictability in the home and have to face family arguments, moves, and changes to new environments. This instability is detrimental to children’s development (Roy et al., 2014; Lawrence et al., 2015). At the same time, due to their young age, their ability to control their surroundings and adjust their state of self is rather limited. If continuously exposed to a chaotic family environment with external environmental stimuli beyond what children can bear, it will increase the pressure on children’s lives (Mollborn et al., 2018). In this regard, children are prone to anxiety, low self-esteem, resentment and other negative emotions and some behavioral problems, which can even lead to extreme difficulty in making strong and optimistic choices in the face of adversity causing children to develop learned helplessness, undermining the development of children’s self-regulatory system, affecting children’s competence, initiative, etc., making it easier for migrant children to compromise or be passive in the face of adversity, which further causes a lower resilience.

### Mediating effect of family resilience

The results found that family resilience mediated the relationship between home chaos and migrant children’s resilience. Home chaos as a risk factor further influenced preschool migrant children’s resilience by affecting family resilience in intra-family psychosocial characteristics. First, how a family organizes itself, how it maintains cohesion, how open it is to communication, and how it works together to solve problems and cope with adversity will largely predict the family resilience (Ungar, 2016). Families of migrant children usually face a noisy, disorganized and disorderly family environment, and these external pressures are easily transformed into psychological conflicts among family members (Marsh et al., 2020), which affect the hindered functioning of family belief systems, organizational systems and communication processes, resulting in family instability (Roy et al., 2014). The chaotic environment tends to lead to family members’ fatigue and affects members’ response and participation, efficacy concept, etc. It is more likely to form low family cohesion and poor structure, which in turn affects the level of family resilience (Marsh et al., 2020). Second, the way in which family uses a variety of resources to cope with the adversity of a chaotic environment affects the way in which individual members are able to cope and adapt (Daniels and Bryan, 2021). Parents who live in a chaotic environment for a long time pay more attention to the external and significant changes of children, but seldom notice the internal changes of children’s resilience, so that children lack timely and reasonable guidance in dealing with problems. Chaotic home environments where parents and children have fewer opportunities for positive and sustained interaction, lack of a good home learning and educational environment, etc., make it difficult for migrant children to receive sufficient care from their families to build good attachments (Whitesell et al., 2015; Hong et al., 2021). For preschool migrant children, family resilience is the most important supportive resource that they can obtain when they encounter difficulties in unfamiliar cities. However, migrant families with low resilience cannot adapt to the external environment and cannot provide appropriate growth environment and supportive resources for migrant children (Daniels and Bryan, 2021). When migrant children encounter difficulties in learning and living, they are blind and helpless, lack initiative and motivation, and believe they lack the ability to solve problems independently. Even when they seek support and help from family members, they do not receive timely support and guidance, which in the long run will lead children to view problems more pessimistically and negatively, doubt their ability to solve problems, and fail to mobilize positive emotions to face difficulties (Westphaln et al., 2022). Conversely, higher family resilience has a significant relationship with migrant children’s resilience development in terms of mutual concern and support among family members, intimate communication, mutual communication, and problem solving, use of socioeconomic resources, maintenance of positive attitudes, and giving meaning to adversity (Chen et al., 2021). Thus, family resilience has a mediating effect on the development of migrant children’s resilience, and family resilience can act as a protective factor in a good state and a risk factor for the development of resilience in migrant children in a bad or disadvantaged situation.

### Moderating effect of social support

The results of this study showed that social support was significantly and positively related to family resilience and significantly and negatively related to home chaos. Social support positively moderated the mediating effects of family resilience in the relationship between home chaos and preschool migrant children’s resilience. It was shown that social support could impair the negative effect of home chaos on family resilience and help to strengthen and foster family resilience (Wong et al., 2019; Hill et al., 2021). When migrant families are under stress or in a chaotic environment, social support can give families the material or spiritual resources they need to help them cope with their problems and achieve positive outcomes (Lei and Kantor, 2021). The social support migrant families receive mainly from family members, friends and significant others. Families with a high level of social support have members who help and support each other and seek internal strength among members. When they encounter difficulties or adversities, they seek support and help from family members, and such inter-member help often gives families enough confidence and strength to face difficulties (Thomas et al., 2017). And when internal strengths cannot be met or conflicts arise among members, families will seek help from more familiar friends, relatives, and neighbors around them or others who are important to seek help. Families get care and help from friends, which can effectively improve their attitudes or abilities when facing difficulties (Meghan et al., 2015; Canton, 2018). Social support as a protective factor of family supportive resources weaken the risky role of family noisiness, and can allow families in chaotic environments to receive help in the form of direct support, emotional support, and provision of advice from within the family, friends and others, etc., which facilitates families to adopt positive coping styles and promote their resilience (Nuri et al., 2020). In addition, the protective effect of social support is more pronounced when home chaos is high, and high social support has an enhancing effect on the protective mechanism of family resilience (Hassanein et al., 2021). When individuals receive appropriate material support and spiritual comfort, they can maintain good family functioning even in relatively high stress environments (Meghan et al., 2015). When feeling the warmth of the family and external support and understanding in migrant families, they are more confident to face external disadvantages and thus better plight and increase the level of resilience. Therefore, it is important to highlight the contribution of social support to the material or moral support given to migrant families which cannot be underestimated.

## Implication

Through the analysis and discussion of the results, it can be concluded that migrant children’s resilience is closely related to home chaos, family resilience and social support in the family environment in which they live. China NPC website (2021), officially implemented in January 2022, clearly defines the responsibilities of families, and points out that we should create a good family environment for children’s development and give full play to the important role of families in promoting children’s healthy growth. Therefore, to help preschool migrant children to improve their resilience, it is necessary to pay attention to the improvement of home chaos, family resilience and social support, so that they could cope with adversity and grow up healthily. For preschool migrant children, the new environment brings great challenges to their physical and mental development, and the chaotic family environment is unfavorable to their physical and mental development (Zhang, 2022). Providing a favorable family environment for preschool children and reducing home chaos is also an important link in protecting and promoting the development of children. Parents of migrant children should pay attention to creating a warm and harmonious family atmosphere, good family rules, a noise-free and suitable for children’s growth of the family environment.

Family resilience is a long-term benign motivation for the development of the resilience of migrant children (Chen et al., 2021). With good family resilience, family members to give timely encouragement and help including providing children with adequate and consistent response, security, family relationship, affect children’s ability to cope with difficulties and solve problems, and provide the resources for individuals in adversity, relieve pressure, promote the individual good adaptation, and reduce an individual’s psychological problems, which will make children form a better resilience (Ungar, 2016; Suzuki et al., 2018). Therefore, the positive role of family resilience should be emphasized, and intervention programs can be constructed on a family basis to play the protective role of family resilience and improve preschool migrant children’s resilience. It should be done to promote positive and effective communication among family members, solve problems together, positively rationalize and evaluate the crisis, and face adversity with an optimistic attitude. It is important to further enhance family cohesion and give full play to the important role of family resilience, which will in turn enhance preschool migrant children’s resilience.

In addition to the need to improve the elements of the family system, a well-functioning family system also requires the cooperation of social support from the external system. A favorable social support system can provide parents with resource support, family education guidance, improve the connection between the family and the outside world, help solve the dilemmas faced in family education, enhance family resilience, and further promote the development of preschool migrant children’s resilience (Barnhart et al., 2022). It should pay attention to building social support systems for migrant families. It is also important to increase social resources for migrant families and help migrant families recognize their situation and identify the strengths, potentials and resources that exist in the family.

## Limitations and future research

With regard to limitations, the data of this study were all from parent reports. Although the common method bias showed no effect of the method, the information obtained may deviate from the real situation. As parents, they may underestimate the home chaos, family resilience and children’s resilience, leading to insufficient objectivity in the results. Therefore, future studies should consider using different kinds of assessment methods, such as adding observational indicators, reported by different information sources, such as children, teachers, and peers, to obtain more objective measurements.

Another limitation concerns, the present study adopted a cross-sectional design, which means that data were collected at a single point in time and could not reveal longitudinal associations between individual variables. Previous studies have shown that home chaos experiences are closely related to children’s future development, and children’s resilience is an evolving process of change. Therefore, future studies should consider collecting longitudinal data to obtain additional findings and extend the research conclusions.

## Conclusion

As the basic psychological quality to cope with adversity, resilience is of great significance to the development of preschool migrant children, and is an important guarantee for their mental health and development. This study, based on resilience model theory, took preschool migrant children in Guangdong Province of China as participants and investigated the relationship and the mechanisms of action between home chaos and preschool migrant children’s resilience. The results showed that, first of all, after controlling for gender and age, home chaos had a negative effect on the development of migrant children’s resilience. Secondly, family resilience played a mediating role in the relationship between home chaos and resilience of preschool migrant children. Finally, social support positively moderated the mediating effects of family resilience. The results of this study help to answer the mechanisms and conditions of home chaos on children’s resilience, and have some practical implications for the development of children’s resilience.

## Data availability statement

The raw data supporting the conclusions of this article will be made available by the authors, without undue reservation.

## Ethics statement

All procedures followed were in accordance with the ethical standards of the responsible committee on human experimentation (Guangzhou University, Guangdong Province, China) and with the Helsinki Declaration of 1975, as revised in 2000. Written informed consent to participate in this study was provided by the participants. The patients/participants provided their written informed consent to participate in this study.

## Author contributions

JZha designed the research and drafted the manuscript. HC collected and extracted data for analysis. HC, JZho, and LZ provided important ideas and substantial feedback for the study and edited the manuscript. All authors contributed to the article and approved the submitted version.

## Funding

This work was supported by the Project of the National Social Science Fund of China (21CSH032).

## Conflict of interest

The authors declare that the research was conducted in the absence of any commercial or financial relationships that could be construed as a potential conflict of interest.

## Publisher’s note

All claims expressed in this article are solely those of the authors and do not necessarily represent those of their affiliated organizations, or those of the publisher, the editors and the reviewers. Any product that may be evaluated in this article, or claim that may be made by its manufacturer, is not guaranteed or endorsed by the publisher.

## Footnotes

^1^https://www.wjx.cn
